# Targeted Therapy for RET Fusion Lung Cancer: Breakthrough and Unresolved Issue

**DOI:** 10.3389/fonc.2021.704084

**Published:** 2021-08-23

**Authors:** Shinkichi Takamori, Taichi Matsubara, Naoki Haratake, Gouji Toyokawa, Takatoshi Fujishita, Ryo Toyozawa, Kensaku Ito, Masafumi Yamaguchi, Kenichi Taguchi, Tatsuro Okamoto, Takashi Seto

**Affiliations:** ^1^Department of Thoracic Oncology, National Hospital Organization Kyushu Cancer Center, Fukuoka, Japan; ^2^Department of Surgery and Science, Graduate School of Medical Sciences, Kyushu University, Fukuoka, Japan; ^3^Department of Thoracic Surgery, National Hospital Organization Kyushu Medical Center, Fukuoka, Japan; ^4^Cancer Pathology Laboratory, National Hospital Organization Kyushu Cancer Center, Fukuoka, Japan

**Keywords:** non-small cell lung cancer, RET, multikinase inhibitor, selective inhibitor, targeted therapy

## Abstract

Molecular drugs targeting mutated or rearranged oncogene drivers have become one of the standard recognized treatments in patients with advanced and recurrent non-small cell lung cancer. *RET* is located in the long arm of human chromosome 10 and encodes a receptor tyrosine kinase protein, and RET fusion-positive lung adenocarcinoma occurs in 1%–2% of cases. Clinical trials of multikinase inhibitors, including cabozantinib, vandetanib, sorafenib, and lenvatinib, that inhibit RET oncogene activity have shown their antitumor efficacy. Recently, RET inhibitors such as pralsetinib and selpercatinib that are specialized for RET kinase have also been developed, and their efficacy was investigated in previous clinical trials (BLU-667 and LOXO-292). In this review, we summarized the effects and adverse events of multikinase and selective RET inhibitors and the various diagnostic techniques for *RET* gene fusion. In the perspective part, we focused on the unsolved issues on treatment for RET fusion-positive lung cancer and future developments.

## Introduction

In recent years, treatment with molecular targeted drugs, focusing on driver genes of advanced and recurrent non-small cell lung cancer, has become one of the standard recognized treatments. In 2012, *RET* fusion gene was identified as a new targetable driver gene (1–3). *RET* is located in the long arm of human chromosome 10 and encodes a receptor tyrosine kinase protein. RET fusion-positive lung adenocarcinoma occurs in 1%–2% of cases, and clinical trials of multikinase inhibitors that inhibit RET oncogene activity such as vandetanib and cabozantinib have indicated their antitumor efficacy. Anticipating the efficacy of precision medicine for RET fusion-positive lung cancer, domestic and international clinical trials of RET inhibitors have been conducted. RET inhibitors such as pralsetinib and selpercatinib that are specialized for RET kinase have been developed, and their efficacy was investigated in several clinical trials.

## Multikinase RET inhibitors

### Cabozantinib

Cabozantinib is a multikinase inhibitor that inhibits specific receptor tyrosine kinase such as RET, vascular endothelial growth factor receptor (VEGFR), mesenchymal–epithelial transition (MET), ROS1, AXL, immunoglobulin-like and epidermal growth factor-like domains 2 (TIE2), and KIT (4). A phase II clinical trial enrolling 26 patients was conducted to determine the efficacy of cabozantinib in metastatic RET fusion-positive lung cancer. The response rate was 28% (95% confidence interval [CI]: 12%–49%), indicating significant efficacy. The report stated that the median progression-free survival was 5.5 months (95% CI: 3.8–8.4 months), and the overall survival was 9.9 months (95% CI: 8.1 months to unreached) (4). Primary adverse events of Grade 3 and higher were elevation in lipase, alanine aminotransferase (AST), and alanine aspartate aminotransferase (ALT) levels, as well as thrombocytopenia and hypophosphatemia, thus necessitating a dosage reduction in 19 patients (73%). The summary of clinical data of RET inhibitors for RET fusion-positive lung cancer is shown in **Table 1**. **Table 2** summarizes clinical trials of RET inhibitors.

**Table 1:** Summary of clinical data of RET inhibitors for RET fusion-positive lung cancer.

RET inhibitors	Multikinase inhibitors												Selective RET inhibitors	
	Cabozantinib		Vandetanib			Sorafenib		Lenvatinib	Sunitinib	Nintedanib	Ponatinib	Regorafenib	Pralsetinib	Selpercatinib
Countries	USA	Global clinical trial (GLORY)	Japan	South Korea	Global clinical trial (GLORY)	Japan	Global clinical trial (GLORY)	Japan, USA, Singapore, Taiwan	Global clinical trial (GLORY)	Global clinical trial (GLORY)	Global clinical trial (GLORY)	Global clinical trial (GLORY)	Global clinical trial	Global clinical trial
Number of cases	26	19	19	17	11	3	2	25	9	2	2	1	Treated: 92 Treatment-naive: 29	Treated: 105 Treatment-naive: 39
Methods of analysis	NGS, FISH (one must be positive)	NGS, RT-PCR, FISH (one must be positive)	RT-PCR, FISH (both must be positive)	FISH, RT-PCR	NGS, RT-PCR, FISH (one must be positive)	RT-PCR, FISH (one must be positive)	NGS, RT-PCR, FISH (one must be positive)	NGS	NGS, RT-PCR, FISH (one must be positive)	NGS, RT-PCR, FISH (one must be positive)	NGS, RT-PCR, FISH (one must be positive)	NGS, RT-PCR, FISH (one must be positive)	NGS, RT-PCR, FISH (one must be positive)	NGS, RT-PCR, FISH (one must be positive)
Fusion partners	*KIF5B* (16 cases), *CCDC6* (1 case), *TRIM33* (1 case), *CLIP1* (1 case), *ERC1* (1 case), other (6 cases)	NA	*KIF5B* (10 cases), *CCDC6* (6 cases), other (3 cases)	*KIF5B* (5 cases), *CCDC6* (2 cases), *MYO5C* (1 case), unknown (10 cases)	NA	*KIF5B* (1 case), *CCDC6* (1 case), unknown (1 case)	NA	*KIF5B* (13 cases), *CCDC6* (12 cases), unknown (1 case)	NA	NA	NA	NA	*KIF5B* (88 cases), *CCDC6* (19 cases), other/unknown (14 cases)	*KIF5B* (59 cases), *CCDC6* (24 cases), *NCOA4* (2 cases), *RELCH* (2 cases), other/unknown (18 cases)
Response rate	28%	37%	53%	18%	18%	0%	0%	16%	22%	50%	0%	0%	Treated: 55% Untreated: 66%	Treated: 64% Untreated: 85%
Progression-free survival (median)	5.5 months	3.6 months	4.7 months	4.5 months	2.9 months	NA	NA	7.3 months	2.2 months	NA	NA	NA	Unreached	Treated: 17 months Untreated: Unreached
Overall survival (median)	9.9 months	4.9 months	11.1 months	11.6 months	10.2 months	NA	NA	NA	6.8 months	NA	NA	NA	Unreached	Unreached
Grade 3 and higher adverse events	47%	NA	58%	28%	NA	33%	NA	92%	NA	NA	NA	NA	NA	28%

GLORY, Global Multicenter RET Registry; NGS, next-generation sequencing; FISH, fluorescence in situ hybridization; NA, not available.

**Table 2:** Summary of clinical trials of RET inhibitors for RET fusion-positive lung cancer.

References	Year	Types and phases	Countries	RET inhibitor	Number of cases	Primary endpoint
Drilon et al. (4)	2016	II	USA	Cabozantinib	26	ORR
Yoh et al. (5)	2017	II	Japan	Vandetanib	19	ORR
Gautschi et al. (6)	2017	Retrospective	Global (Europe, Asia, and the USA)	Cabozantinib, vandetanib, sunitinib, sorafenib, alectinib, lenvatinib, nintedanib, ponatinib, regorafenib	165	–
Lee et al. (7)	2017	II	South Korea	Vandetanib	18	ORR
Hida et al. (8)	2019	II	USA, Japan, Singapore, and Taiwan	Lenvatinib	25	ORR
Horiike et al. (9)	2016	II	Japan	Sorafenib	3	–
Gainor et al. (10)	2020	I/II	Global (11 countries)	Pralsetinib	121	ORR
Drilon et al. (11)	2020	I/II	Global (12 countries)	Selpercatinib	144	ORR

ORR, objective response rate.

### Vandetanib

Vandetanib is another multikinase inhibitor that inhibits RET, VEGFR, and epidermal growth factor receptor (EGFR) signaling (5). A phase II investigator-initiated clinical trial (LURET) enrolling 19 patients was conducted in Japan to determine the efficacy of vandetanib in metastatic RET fusion-positive lung cancer. The response rate was 53% (95% CI: 28%–77%), indicating significant efficacy. The median progression-free survival was 4.7 months (95% CI: 2.8–8.5 months) (5). In addition, 11 retrospective studies conducted by the Global Multicenter RET Registry (GLORY) showed the response rate to be 18% and the median progression-free survival to be 2.9 months. In the GLORY study, the median line of systemic therapy of the first RET inhibitors administered was third line, ranging from first to eighth. Primary adverse events included diarrhea, rash, hypertension, and asymptomatic long QT syndrome. Due to these adverse events, dosage reduction was imperative in 20% of the targeted patients, and the treatment was discontinued in 50% of them (6). A similar phase II clinical trial enrolling 17 patients was also conducted in South Korea. The response rate was 18%, and the median progression-free survival was 4.5 months (7). Based on this result, the NCCN Guideline (Version 3; 2020) listed vandetanib under Category 2A as an appropriate treatment for metastatic RET fusion-positive lung cancer (12).

### Lenvatinib

Lenvatinib inhibits RET, VEGFR, platelet-derived growth factor receptor (PDGFR), fibroblast growth factor (FGF), and c-KIT (8). Phase II clinical trials enrolling a total of 25 patients were conducted to determine the efficacy of lenvatinib in metastatic RET fusion-positive lung cancer patients in Japan, the United States, Singapore, and Taiwan. The response rate was 16% (95% CI: 12%–49%), and the median progression-free survival was 7.3 months (95% CI: 3.6–10.2 months) (8). Adverse events of Grade 3 and higher were observed in 92% of targeted patients and included hypertension, nausea, vomiting, anorexia, diarrhea, and proteinuria. Dosage reduction was imperative in 64% of targeted patients, and treatment was discontinued in 20% of the targeted patients due to these conditions.

### Other Multikinase Inhibitors

Retrospective studies conducted by GLORY revealed therapeutic response in 2/9 sunitinib (inhibitor of RET, VEGFR, PDGFR, c-KIT, and fms-related tyrosine kinase 3 [FLT3]) cases, 1/2 nintedanib (inhibitor of RET, VEGFR, PDGFR, and FGFR) cases, 0/2 ponatinib (inhibitor of RET, VEGFR, PDGFR, FGFR, c-KIT, and Src) cases, 0/2 sorafenib (inhibitor of RET, VEGFR, PDGFR, FLT3, and KIT) cases, and 0/1 regorafenib (inhibitor of RET, VEGFR, FGFR, PDGFR, c-KIT, and TIE2) case (6). A phase II clinical trial enrolling three patients was conducted to determine the efficacy of sorafenib in metastatic RET fusion-positive lung cancer. In this trial, one patient exhibited stable disease, and two patients exhibited progressive disease (9).

## Selective RET inhibitors

### Pralsetinib

Pralsetinib (BLU-667) is a highly selective RET inhibitor. Pralsetinib inhibits wild-type RET and oncogenic RET fusions (KIF5B-RET, CCDC6-RET, etc.) and mutations (V804L, V804M, and M918T). The ARROW clinical trial (Phase 1/2) reported that the response rate of pralsetinib in treatment-naive RET-altered non-small cell lung cancer patients was 73% (95% CI: 52%–88%), and the response rate in treated patients was 61% (95% CI: 50%–72%) (10). The median follow-up period was 8.8 months, and progression-free survival was not reached. Approximately 50% of the RET-altered non-small cell lung cancer patients were found to have developed brain metastasis, but the intracranial responses of pralsetinib that were tested in the patient group with brain metastasis revealed a satisfactory response rate of 56%. A low occurrence rate of 6.9% for Grade 3 adverse events such as elevation in ALT level, tumor lysis syndrome, and hypertension was reported. No Grade 4 or 5 adverse events were recognized. The U.S. Food and Drug Administration approved pralsetinib in September 2020 and designated it as a breakthrough therapy.

### Selpercatinib

Selpercatinib (LOXO-292), similar to pralsetinib, is a highly selective RET inhibitor. Selpercatinib inhibits wild-type RET and oncogenic RET fusions (KIF5B-RET, CCDC6-RET, etc.) and mutations (V804L, V804M, and M918T). The LIBRETTO-001 clinical trial (Phase 1/2) enrolled 39 treatment-naive patients and 105 patients previously treated with platinum-based chemotherapy (11). In this clinical trial, the response rate of selpercatinib in untreated patients was 85% (95% CI: 70%–94%), and progression-free survival was not reached. The response rate of selpercatinib in pretreated and untreated patients was 64% (95% CI: 54%–73%) and 85% (95% CI: 70%–94%), respectively. The median progression-free survival was 17 months (95% CI: 14 months to unreached). These pretreated patients had previously received multiple treatments (the median number of prior treatment lines was 3; 55% had received anti-PD-1/PD-L1 antibodies; and 48% had received at least one multikinase inhibitor); however, the response rate did not significantly differ between untreated and pretreated patients regardless of previous treatments. The intracranial response of selpercatinib showed a satisfactory response rate of 91% (95% CI: 59%–100%). Primary adverse events included diarrhea, general malaise, and xerostomia. Forty-one cases of treatment-related Grade 3 and higher adverse events out of 144 (28%) were reported, including tumor lysis syndrome, elevation in AST/ALT levels, diarrhea, and thrombocytopenia. The U.S. Food and Drug Administration approved selpercatinib in May 2020 and designated it as a breakthrough therapy along with pralsetinib. The response rate of selpercatinib was 60% (95% CI: 43%–75%) in a subgroup of East Asian patients (n = 40), and the results were announced at the 2021 World Conference of Lung Cancer, based on which the therapeutic effects of selpercatinib in East Asians were adjudged to be equal to those in Europeans and Americans.

## Perspective

### Unsolved Issues on Treatment for RET Fusion-Positive Lung Cancer and Future Developments

1) Based on the data above, selective RET inhibitors could be considered for future clinical trials. The progression-free survival of RET fusion-positive lung cancer patients treated with multikinase inhibitors including sunitinib, cabozantinib, and vandetanib was reported as 2.2–4.7 months. Conversely, the progression-free survival for selpercatinib, a selective RET inhibitor, was 17 months, even in pretreated patients. The difference in the progression-free survival could be attributed to the difference in the IC50 values of multikinase inhibitors and selective RET inhibitors (13). For instance, the IC50 value of vandetanib for V804M is 76 nM, but that of selpercatinib is 0.8 nM, which is many folds lower. V804M is a RET inhibitor-resistant gatekeeper mutation, which confers a gain of function on the RET protein. Hypothetically, even if the dose of vandetanib was increased to increase its blood concentration to inhibit V804M, VEGFR2 is forcefully inhibited, thus increasing toxicity. Perhaps in future clinical trials of RET fusion-positive lung cancer, selective RET inhibitors will become mainstream.
2) Recruitment of appropriate patients for determining examination methods in RET fusion-positive lung cancer is crucial. The LURET clinical trial required participation of patients who tested positive by both RT-PCR and fluorescence *in situ* hybridization (FISH) methods (5). In a clinical trial that was performed in South Korea, RET rearrangements were detected by FISH and further confirmed by immunohistochemistry, RT-PCR, or targeted deep sequencing-based panel assay in cases with available study materials (7). A recent study indicated that the diagnostic sensitivity of RET fusion-positive lung cancer using the FISH technique was 100% (85.8%–100%), and the specificity was 85% (62.1–96.8%), although the specificity may be underestimated given that the study set the positivity cutoff to be ≥10% tumor cells, demonstrating a *RET* rearrangement pattern (14). Analysis of lung and thyroid cancer tissues showed a positive rate by RET fusion partners such as *KIF5B* and *CCDC6*, and their sensitivities were 100%. However, the sensitivity for NCOA4 was 66.7% (34.9%–90.1%), although the study investigated small sample size (12 cases) and definitive conclusions cannot be drawn at present (14). The FISH technique may not be accurate for certain fusion partners. Furthermore, the LIBRETTO-001 and ARROW trials accepted patients who tested positive for RET fusion using methods that were utilized at each local facility (next-generation sequencing (NGS), RT-PCR, or FISH). Neither trials required a central confirmatory diagnosis. Thus, definitive methods for detecting RET fusion-positive lung cancer need to be established.
3) In recent years, treatment with immune checkpoint inhibitors, alone or in combination with chemotherapy, has become standard for advanced and recurrent non-small cell lung cancer; therefore, treatment regimens for RET fusion-positive lung cancer patients must be carefully considered. Some RET fusion-positive lung cancer patients reported from their retrospective perspective that targeted treatment is preferable over treatment with immune checkpoint inhibitors for better prognosis (15). Moreover, previous studies indicated that the use of immune checkpoint inhibitors increases the risk of drug-induced pneumonitis if various tyrosine kinase inhibitors such as EGFR-TKIs were previously administered (16). Therefore, the safety of using immune checkpoint inhibitors after administering RET inhibitors is concerning. Previous studies also showed that RET fusion-positive lung cancer patients may benefit a lot from pemetrexed-based treatments (17); therefore, treatment regimens that include immunotherapy and chemotherapy must be considered. A randomized phase III trial is currently ongoing for RET fusion-positive lung cancer patients to determine the efficacy of combination therapy with selective RET inhibitors and chemotherapy ± pembrolizumab (18, 19). The results of this trial may facilitate development of better treatment regimens.
4) It is crucial to thoroughly comprehend the mechanisms of resistance for RET fusion-positive lung cancer because resistance is inevitable even in cases of initial success after administrating an RET inhibitor. In a previous study, analysis of the biopsy specimens of patients after they received selective RET inhibitors (n = 18) revealed secondary acquired RET mutations in two cases (G810), acquired MET amplification in three cases, and acquired KRAS amplification in one case. It also reported squamous or small-cell histologic transformation (20). Further research on RET inhibitor resistance and development of drugs to overcome the resistance is needed.
5) The LIBRETTO-001 trial involved a subgroup analysis of response rates considering various fusion partners such as *KIF5B*, *CCDC6*, and *NCOA4* and reported that response rates tended to be higher with the *11CCDC6* fusion partner. Because of insufficient examination due to the low frequency of RET fusion gene, further research in a larger sample size considering various treatment strategies based on fusion partners is expected.
6) RET fusion-positive lung cancer is often followed by brain metastasis, but among previously enrolled patients in clinical trials, very few were diagnosed with brain metastasis; thus, there is a lack of sufficient data concerning brain metastasis at this moment. The LIBRETTO-001 and ARROW trials had 11 and nine RET fusion-positive lung cancer patients, respectively, who also had brain metastasis (10, 11). Further examination of the response rates for RET fusion-positive lung cancer and treatment strategies for symptomatic brain metastases is critically needed.
7) Global clinical trials for these rare lung cancers are more beneficial than domestic trials, because global trials allow faster case registration and delivery of faster positive results, thus increasing the number of patients who are benefitted. Furthermore, the bias toward domestic treatment policies can also be diminished.

## Conclusions

Since the discovery of RET fusion gene as a new driver gene for lung cancer in 2012, numerous clinical trials have been conducted, and many highly efficacious selective RET inhibitors have been developed. For RET fusion-positive lung cancer patients, this is good news. Pursuing various methods of RET fusion testing, treatment regimens, response results involving various fusion partners, and overcoming drug resistance will allow development of ideal treatment strategies for RET fusion-positive lung cancer patients.

## Author Contributions

ST wrote the manuscript. TM and NH collected previous articles. GT and MY significantly contributed to the methodology. TF, RT, and KI significantly contributed to all of the ideas of the current article. KT, TO, and TS supervised the writing of the manuscript. All authors contributed to the article and approved the submitted version.

## Conflict of Interest

As a potential personal and financial conflict of interest, RT reports personal fees from Kyowa Hakko Kirin, Nippon Kayaku, and Novartis Pharma; and grants from Abbvie, Daiichi Sankyo, Pfizer Japan, and Takeda Pharmaceutical. MY reports personal fees from AstraZeneca, Nippon Boehringer Ingelheim, Ono Pharmaceutical, and Taiho Pharmaceutical; and grants from Chugai Pharmaceutical and Pfizer Japan. TS reports grants from Bayer Yakuhin, Eisai, Merck Serono, and Novartis Pharma; personal fees from Bristol-Myers Squibb, Kyowa Hakko Kirin, Mochida Pharmaceutical, Ono Pharmaceutical, Roche Singapore, Showa Yakuhin, Taiho Pharmaceutical, and Takeda Pharmaceutical; and grants and personal fees from Astellas Pharma, AstraZeneca, Chugai Pharmaceutical, Daiichi-Sankyo, Eli Lilly Japan, Kissei Pharmaceutical, MSD, Nippon Boehringer Ingelheim, Pfizer Japan, and Yakult Honsha, outside the submitted work.

The remaining authors declare that the research was conducted in the absence of any commercial or financial relationships that could be construed as a potential conflict of interest.

## Publisher’s Note

All claims expressed in this article are solely those of the authors and do not necessarily represent those of their affiliated organizations, or those of the publisher, the editors and the reviewers. Any product that may be evaluated in this article, or claim that may be made by its manufacturer, is not guaranteed or endorsed by the publisher.
